# A retrospective study on factors associated with COVID-19 vaccine refusal among individuals aged 15 years and above in the general population of Bamenda I sub-division, Cameroon

**DOI:** 10.3389/fpubh.2025.1539459

**Published:** 2025-10-02

**Authors:** Yamssi Cédric, Noumedem Anangmo Christelle Nadia, Abongwa Delight Chewah, Wenjuan Liu, Ye Liu, Tako Djimefo Alex Kevin, Vincent Khan Payne, Haibo Hu

**Affiliations:** ^1^Department of Biomedical Sciences, Faculty of Health Sciences, University of Bamenda, Bambili, Cameroon; ^2^Laboratory of Tropical and Emerging Infectious Diseases, Yaoundé, Cameroon; ^3^Department of Microbiology, Haematology and Immunology Faculty of Medicine and Pharmaceutical Sciences, University of Dschang, Dschang, Cameroon; ^4^Department of Public Health, Faculty of Health Sciences, University of Bamenda, Bambili, Cameroon; ^5^Jiangxi Province Key Laboratory of Pharmacology of Traditional Chinese Medicine, National Engineering Research Center for Modernization of Traditional Chinese Medicine-Hakka Medical Resources Branch, School of Pharmacy, Gannan Medical University, Ganzhou, China; ^6^Department of Animal Organisms, Faculty of Science, University of Douala, Douala, Cameroon; ^7^Department of Animal Biology, Faculty of Science, University of Dschang, Dschang, Cameroon

**Keywords:** COVID-19, COVID-19 vaccine, vaccine hesitancy, Bamenda I, vaccine misinformation

## Abstract

**Background:**

By the end of 2020, several vaccines aimed at combating COVID-19 were authorized for widespread immunization. The aim of this study was to assess the factors that were associated with COVID-19 Vaccine refusal in Bamenda I.

**Method:**

This was a retrospective study carried out in the subdivision of Bamenda I, from March to June 2024, through interviews and questionnaires in which participants were asked to recall important events during the COVID-19 pandemic. A questionnaire-based survey was conducted to ensure diverse representation in the community study. The questionnaire comprised four sections: socio-demographic characteristics, knowledge about COVID-19 and vaccines, attitudes toward COVID-19, and beliefs about the vaccine. Data were collected during community gatherings, such as focus group discussions and training sessions, and also through Google Forms via the Kobo tool for participants with Android phones. Data were then transferred to SPSS for analysis, using the Pearson chi-square test to examine associations, with *p*-values <0.05 deemed significant.

**Results:**

Our results show that 42.40% participants had high knowledge about COVID-19, 39.40% had moderate knowledge while 18.20% had low knowledge. Their vaccination status, showed that, just 12.10% were vaccinated, and 87.90% were unvaccinated. Socio-demographic factors like age, gender, and education recorded significant associations with respect to vaccine refusal *p*-value < 0.05. Participants of the study (80.8%) were concerned about the potential side effects of vaccines, while 19.2% said they were not. Some participants (53.5%) mentioned that vaccines were not necessary now that the pandemic has slowed down, while 43.5% disagreed. When asked if they will advise their family and friends to get vaccinated, 9.1% strongly agreed, 21.2% agreed, 40.4% were neutral, 17.2% disagreed, and 12.1% strongly disagreed. A total of 17.2% had underlying health conditions which prevented them from receiving the vaccine while 82.8% had no underlying health conditions. Many believed that the vaccines were promoted for financial reasons. Also, when asked if they knew some preventive measures of the disease, 17.2% said taking COVID-19 vaccine could help in prevention, 13.1% said hand washing could help, 17.2% said wearing of nose masks, 14.1% believed herbal remedies were most suitable and a majority, 38.4% believed in the combination of all of the above measures to prevent the disease.

**Conclusion:**

The study highlights significant knowledge gaps and widespread concerns about vaccine safety among the population in Bamenda I, indicating a need for targeted educational interventions to improve vaccination rates.

## 1 Introduction

The severe acute respiratory syndrome coronavirus-2 (SARS-CoV-2) is the agent behind coronavirus disease 2019 (COVID-19), which surfaced in late 2019 and has caused over 5 million deaths and more than 270 million confirmed cases (1). The virus was first identified in December 2019 in Wuhan, Hubei province, China, following a cluster of pneumonia cases of unknown origin (2). It quickly spread globally, leading the World Health Organization (WHO) to declare it a Public Health Emergency of International Concern on January 30, 2020, and later a pandemic on March 11, 2020.

The WHO’s Strategic Preparedness and Response Plan 2021 sought to address the COVID-19 pandemic by strengthening national health systems’ abilities to detect, prevent, and treat the virus (3). Governments and health systems worldwide responded with measures such as lockdowns, social distancing, mask mandates, and mass vaccination campaigns (4). Several vaccines, including mRNA-based and viral vector vaccines, were developed and authorized for emergency use within a year an unprecedented achievement in medical science. The COVAX facility aims to guarantee worldwide access to safe and effective COVID-19 vaccines. As of November 2021, 7.8 billion vaccine doses had been administered worldwide, with Africa receiving 227 million doses (5). COVID-19 has had profound social, economic, and psychological impacts worldwide. It has also spurred major advancements in public health infrastructure, vaccine technology, and global health cooperation (6). As of recent years, the disease has transitioned from a pandemic to an endemic stage in many regions, with ongoing surveillance and vaccination efforts aimed at controlling its spread and impact (7).

COVID-19 vaccine refusal, which differs from vaccine hesitancy, is the deliberate and steadfast unwillingness to vaccinate despite the availability of safe vaccines (8). While hesitancy is defined by delay or indecision, refusal is defined by adamant refusal (9). In most of the African contexts, for instance in Cameroon, refusal of vaccine has been attributed to several factors ranging from doubt in government and public health organizations, concern over side effects, religious or cultural motivations, and dissemination of misinformation and conspiracy theories, most notably through social media (10). Additionally, the perception that COVID-19 poses a localized limited threat has led to lower urgency perception, further contributing to refusal (11). These are more likely based on more general structural and historical concerns, including previous negative encounters with health care systems.

However, despite improvements in vaccine availability, significant hesitancy toward COVID-19 vaccination has hindered efforts to achieve higher immunization rates (12). COVID-19 reached the African continent relatively later than in Asia, Europe, or the Americas. The first confirmed case in Africa was reported in Egypt on February 14, 2020, followed by a rapid spread across the continent (13). Africa’s initial response was marked by swift governmental actions, including border closures, curfews, and public health campaigns, which helped delay the widespread transmission in the early months (14). Despite fears that the continent’s fragile health systems could be overwhelmed, Africa experienced lower reported infection and mortality rates compared to other continents in the initial phases of the pandemic (15). Several factors contributed to this, including a younger population, experience with epidemic responses (e.g., Ebola), and community-based health structures. However, limited testing capacity, underreporting, and disparities in access to health care and vaccines posed significant challenges. Africa exhibited a diverse vaccination uptake, ranging from 6.9 to 97.9% across the continent (16). The prevalence and mortality rates of COVID-19 vary widely among populations due to several factors, including adherence to containment measures, the reliability of diagnostics and reporting systems, demographics, climate, environmental influences, genetic factors, and immunological variations (17). By the end of 2020, several vaccines aimed at combating COVID-19 were expected to be authorized for widespread immunization.

The first confirmed case in Cameroon was a French national who arrived in Yaoundé on March 6, 2020. Since then, various preventive measures have been implemented nationwide, including reducing public transit use, caring for and quarantining those infected or suspected of infection, banning gatherings larger than 50 people, regulating consumer flow in markets and supermarkets, conducting virtual meetings, avoiding physical contact like handshakes, and covering mouths while sneezing (18). Cameroon’s nationwide vaccination program started on April 12, 2021, utilizing Sinopharm and AstraZeneca vaccines, aiming to vaccinate at least 15 million people (19). The COVID-19 vaccination was to reduce disease-related disability and mortality by controlling the transmission and severity of the SARS-CoV-2 virus.

Additionally, protecting vulnerable populations who cannot be vaccinated is crucial through achieving herd immunity. Herd immunity has successfully eradicated smallpox and other deadly infectious diseases (20). By February 2022, 6.5% of the population in Cameroon, as well as 11.9% in Nigeria had administered at least one dose of COVID-19 vaccine (21). Despite these efforts, Cameroon faced challenges such as limited testing capacity, insufficient health infrastructure, and vaccine hesitancy. Health workers played a vital role in community outreach and awareness campaigns (22). The national COVID-19 vaccination campaign began in April 2021, using vaccines such as Sinopharm, AstraZeneca, Johnson & Johnson, and later Pfizer-BioNTech. However, vaccine uptake was slow due to misinformation, skepticism, and logistical constraints (23). By the end of 2022, Cameroon, like many African countries, had transitioned to a more targeted approach focusing on vulnerable populations and integrating COVID-19 management into routine healthcare services (24). The pandemic also highlighted the importance of strengthening health systems, digital health surveillance, and public health preparedness in Cameroon.

Studying COVID-19 vaccine refusal in Bamenda I, Cameroon, is important due to the underlying socio-political, cultural, and public health environment. Bamenda I is situated within a conflict area in the Northwest Region, where government suspicion, poor healthcare access, and misinformation spread are increased (25, 26). These conditions can result in low vaccine uptake, but data on refusal trends are scarce. Understanding the specific drivers of refusal in this setting is needed to develop context-appropriate public health responses, promote vaccine use, and prevent possible future outbreaks not only of COVID-19 but also of other preventable infectious diseases (27). Results from this local research would contribute to general principles guiding risk communication and trust building in health systems for similarly affected communities. The aim of this study was to assess the factors associated with COVID-19 vaccine refusal among individuals aged 15 years and above in Bamenda I Sub-Division, Cameroon.

## 2 Materials and methods

### 2.1 Study design

This was a retrospective study carried out in the subdivision of Bamenda I, from March to June 2024, through interviews and questionnaires in which participants were asked to recall important events during the COVID-19 pandemic.

### 2.2 Study area

**Figure 1:**
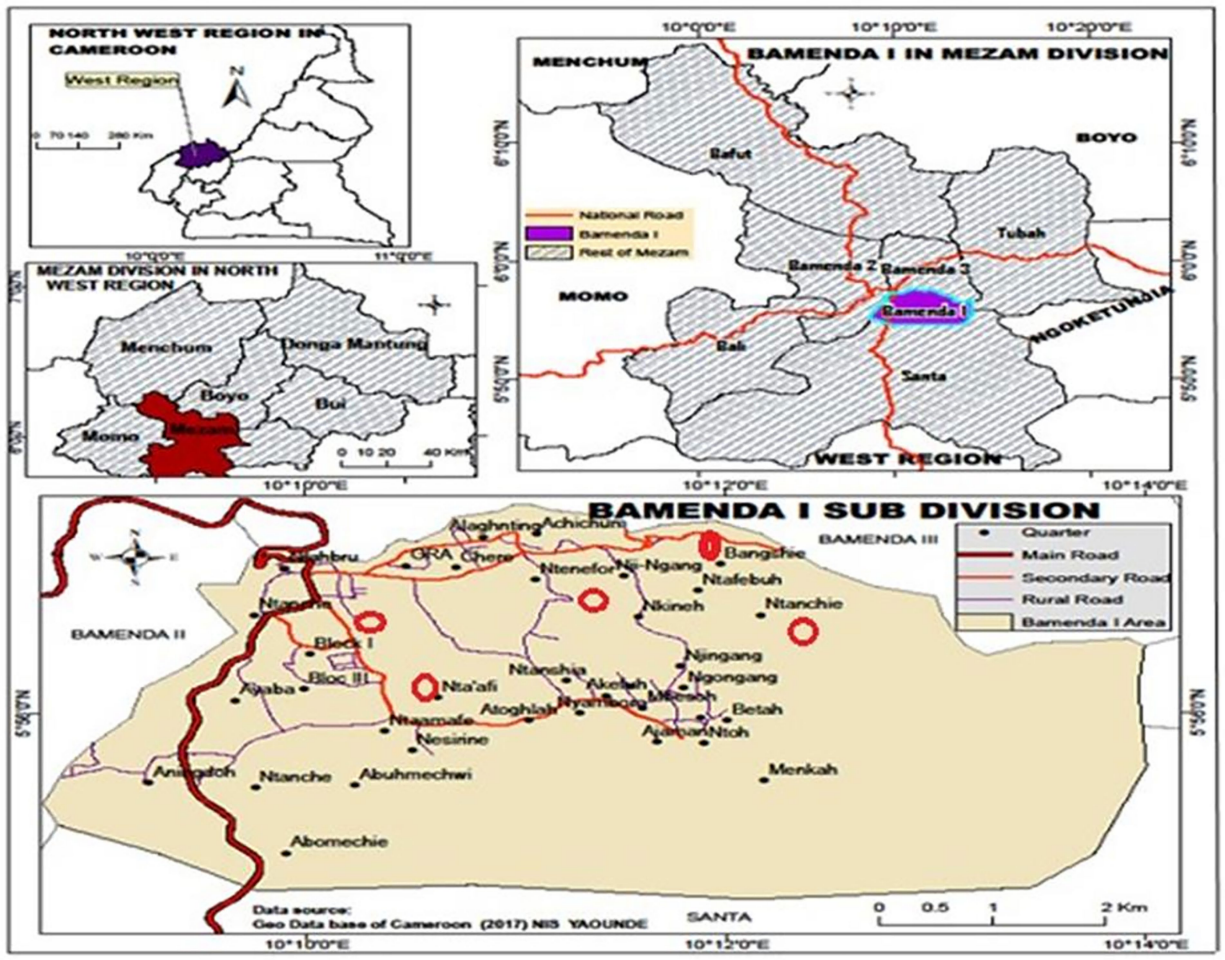
Map of Bamenda I sub-division.

The Bamenda I community served as the research study’s current setting. In the Mezam department of the North-West Region of Cameroon, the urban town of Bamenda contains the district commune known as Bamenda I, or Bamenda I as seen on Figure 1. The district of Bamendankwe serves as its capital. One of the three districts that make up the Bamenda Urban Community is the district commune of Bamenda I, which was established in 2007. According to the 2005 census, there were 28,359 people, with 18,468 living in Bamenda Town’s urban area. The three major ethnic groups are the Bamendankwe, Bamilékés, and Bafut. The town is home to four health facilities: a military hospital and camp, the Bamendankwe Health Center in Akefu, the Bamenda Polyclinic Station in Alatining, and the World Hospital in Ayaba.

### 2.3 Sample size determination

The sample size was calculated using Lorenz’s formula (StatCalc of EPI Info software). Using the prevalence of 10% of a study on the factors associated with COVID-19 vaccine refusal: A community-based study in the Menoua division in Cameroon (28), with an 80% power to detect significant associations or differences and a 5% accepted margin of error, the minimal sample size estimate was 139 participants.

The sample size was calculated using the Lorentz’ formula which is expressed as follows:


$$n=\frac{z^{2}\times p\times (1-p)}{d^{2}}$$

Where:

Z = 1.96 (for 95% confidence level).

*p* = 0.10 (10% prevalence).

d = 0.05 (5% margin of error).

Substituting into the formula:


$$\begin{array}{l} n={\left(1.96\right)}^{2}\times 0.10\times (1-0.10)/{\left(0.05\right)}^{2}\\ =3.8416\times 0.10\times 0.90/0.0025\\ =0.345744/0.0025\\ =138.3 \end{array}$$

Therefore, the minimum required sample size is approximately 139 participants (after rounding up).

#### 2.3.1 Sampling technique

A convenience sample method was used, as it is the most suitable selection method for this study.

#### 2.3.2 Inclusion and exclusion criteria

Everyone from 15 years and above, including those who refused to be vaccinated against COVID-19 and were willing to participate were included in the study. Meanwhile, those with incoherent data or incompletely filled questionnaires were excluded. Also, questionnaire was structured based on specific objectives.

### 2.4 Data collection procedure

A pretest was carried out in the Bamenda I sub-division, involving 10 randomly selected residents aged 15 years and above. The questionnaire was given to them, and following the pretest, complex questions were simplified for easier understanding, leading to the validation of the questionnaire. A questionnaire-based survey was employed in this community-based study to ensure representation across all population categories. The questionnaire was divided into four sections: socio-demographic characteristics, knowledge about COVID-19 and vaccines, attitudes toward COVID-19, and beliefs regarding the COVID-19 vaccine. The first section gathered information on age, gender, education level, and profession, with participants responding by marking the letter that matched their answer. Most questions were closed-ended to facilitate comparison. The second section assessed participants’ knowledge about COVID-19, including the virus itself, preventive measures, awareness of local vaccination centers, concerns about vaccine side effects, and any underlying health conditions that might affect vaccination eligibility. The third section explored whether participants would recommend the vaccine to family and friends, if they would get vaccinated for travel purposes, whether they would cease preventive measures after their first dose, and if they would discuss vaccine risks and benefits with healthcare professionals while pregnant or planning to conceive. Responses were rated on a scale from “Strongly agree” to “Strongly disagree.” The fourth section included questions about whether the COVID-19 vaccine should be administered to older adults, concerns regarding its safety for women planning to conceive, and whether financial motives drive vaccine promotion.

Data collection took place during community gatherings events, including church meetings, health education sessions, market-day gatherings, focus group discussions, and training workshops. These settings provided convenient and trusted spaces within which to invite participants into open discussion about COVID-19 vaccine refusal. Additionally, Google Forms were administered via Kobo Collect to participants with access to Android phones. For those facing language or literacy barriers, we filled out the response sheets after obtaining verbal consent. Participants were informed that their personal information would be kept confidential and that results might be published for academic purposes.

### 2.5 Measurement of COVID-19 vaccine refusal

COVID-19 vaccine refusal was assessed through a structured questionnaire administered to participants aged 15 years and above residing in Bamenda I Sub-Division.

- Participants were asked the direct question: *“Have you received the COVID-19 vaccine?”*

 o Those answering “Yes” were classified as *vaccinated*.
 o Those answering “No” were further asked: *“If no, what is the main reason you have not been vaccinated?”*

- Respondents who explicitly stated lack of trust in the vaccine, disbelief in COVID-19, fear of side effects, or outright unwillingness to ever receive the vaccine were categorized as *refusers*.
- Respondents citing temporary reasons (e.g., illness, waiting for availability, lack of time, or intention to vaccinate later) were classified as *delayers/hesitant*.

Associated factors: Socio-demographic characteristics (age, sex, marital status, education, occupation), health status (history of chronic illness, previous COVID-19 infection), and attitudinal factors (knowledge about COVID-19, perception of vaccine safety and effectiveness, trust in health authorities, and exposure to misinformation) were also measured and analyzed for their association with vaccine refusal.

### 2.6 Ethical considerations

Prior to data collection, ethical clearance was obtained from the Faculty of Health Science (FHS) at the University of Bamenda, with a project identification number (2024/0108H/Uba/IRB). Administrative authorization was also obtained from the North West Regional Delegation of the Ministry of Public Health of Cameroon, the District Health Services, and the directors of the hospitals. During data collection, informed consent was obtained from all participants. For literate individuals, the purpose of the study and their rights were explained verbally, and their oral consent was recorded before beginning the questionnaire. For participants with limited literacy, field workers provided detailed verbal explanations in the participant’s preferred language, ensuring they fully understood the study before consenting. In cases involving minors aged 15–17 years, verbal assent was obtained from the participant along with verbal consent from a parent, guardian, or responsible adult present at the time. All participants were assured that their responses would remain anonymous and confidential, and that their personal data would not be shared. The results would be used strictly for academic research and public health improvement purposes.

### 2.7 Statistical analysis

The data were subsequently transferred to the Statistical Package for Social Sciences (SPSS) software for analysis. Frequency distribution tables were generated for categorical variables, and the Pearson chi-square test was conducted to assess associations between variables. *p* < 0.05 were considered statistically significant. Knowledge levels regarding COVID-19 vaccines were categorized into three groups based on participant scores from an ordinal scale. Participants scoring 8 or higher were classified as having a high level of knowledge, those scoring exactly 5 were deemed to have a moderate level, and scores of 3 or 4 indicated a low level of knowledge.

## 3 Results

### 3.1 Socio-demographic characteristics of the study population

Table 1 shows the socio-demographic data of participants. The study consisted of 198 respondents who were partitioned according to different age groups which ranged from 15 years and above, who were either male or female. It included adolescents who made up 44.4% of the study population, followed by the young adult population who made up 33.3%, followed by the old adult population who accounted for 17.2%, and finally the oldest population made up 5.1% of the total respondents. It also recorded 47.5% male, and 52.5% female respondents. Two percent of respondents had attained just primary education, 26.3% ended at the level of secondary education, 58.6% had completed their university education, and 13.1% were uneducated. 20.2% respondents were entrepreneurs, 9.1% were health personnel, 49.5% were students, and 21.2% were involved in other professions.

**Table 1:** Socio-demographic data.

Variable	Modality	Frequency	Percentage
Age	15–20	88	44.4
	21–30	66	33.3
	31–40	34	17.2
	40 and above	10	5.1
Gender	Male	94	47.5
	Female	104	52.5
Education	Primary	4	2.0
	Secondary	52	26.3
	University	116	58.6
	Not educated	26	13.1
Profession	Entrepreneur	40	20.2
	Health personnel	18	9.1
	Student	98	49.5
	Others	42	21.2

### 3.2 Knowledge of respondents on COVID-19

Table 2 illustrate the respondents’ knowledge about COVID-19. When asked about the virus causing COVID-19, 2.0% identified it as the Ebola virus, 94.9% correctly named the coronavirus, 6% mentioned Hepatitis A. Regarding the availability of vaccination centers during the peak of the pandemic, 20.2% reported yes, 29.3% said no, and 50.5% were unsure. As for reasons for vaccine refusal, 34.3% thought it was dangerous to their health, 11.1% were restricted by their parents, 6.1% cited religious objections, and 48.5% had no specific reasons. When asked about preventive measures, 17.2% mentioned that the COVID-19 vaccine could help, 13.1% believed hand washing was effective, another 17.2% highlighted wearing masks, 14.1% preferred herbal remedies, and the majority, 38.4%, supported using a combination of all these measures for prevention.

**Table 2:** Knowledge of respondents on COVID-19.

Variable	Modality	Frequency	Percentage
Virus responsible for COVID-19 infection?	Ebola	4	2.0
	Corona	188	94.9
	Hepatitis A	6	3.0
	Herpes simplex	0	0.0
Awareness of COVID-19 vaccination center nearby?	Yes	40	20.2
	No	58	29.3
	I do not know	100	50.5
Main reason for COVID-19 vaccine Refusal	Vaccine may be dangerous	68	34.3
	Parents refused	22	11.1
	Religion forbids me	12	6.1
	I do not have a reason	96	48.5
Safety concerns about COVID-19 vaccine?	Yes	156	78.8
	No	42	21.2
Believed in effectiveness of vaccine?	Yes	88	44.4
	No	110	55.6
COVID-19 is a serious public health threat?	Yes	148	74.7
	No	50	25.3
Preventive measures against COVID-19?	Covid 19 vaccine	34	17.2
	Hand washing	26	13.1
	Nose masks	34	17.2
	Herbal remedies	28	14.1
	All of above	76	38.4
COVID-19 vaccine development was rushed?	Yes	100	50.5
	No	98	49.5
Encountered misinformation about the COVID-19 vaccine?	Yes	106	53.5
	No	92	46.5

Table 3 assess the level of knowledge respondents had that could influence their reasons for vaccine refusal. When asked if respondents had underlying health conditions that could have prevented them from receiving the vaccine, 17.2% said yes while 82.8% said no. When asked their thoughts about natural immunity and vaccine-induced immunity, 65.7% chose natural immunity, while 34.3% chose vaccine-induced immunity. On finding out if they saw no necessity now that the pandemic has slowed down, 53.5% said yes, while 46.5% disagreed.

**Table 3:** Knowledge of participants about COVID-19 vaccine.

Variable	Modality	Frequency	Percentage
Any underlying health conditions?	Yes	34	17.2
	No	164	82.8
COVID-19 vaccine is a personal choice or a public health responsibility	Personal choice	129	65.2
	Public health responsibility	69	34.8
Worried about potential side effects of the COVID-19 vaccine?	1	160	80.8
	2	38	19.2
Believed that natural immunity is better than vaccine-induced immunity?	Yes	130	65.7
	No	68	34.3
Concerned about the long-term effects of the COVID-19 vaccine?	Yes	136	68.7
	No	62	31.3
COVID-19 vaccine is unnecessary now that the pandemic has slowed down?	Yes	106	53.5
	No	92	46.5
Heard about any potential religious or ethical concerns related to the COVID-19 vaccine?	Yes	84	42.4
	No	114	57.6
Did vaccine hesitancy contribute to the spread of COVID-19?	Yes	80	80.4
	No	118	59.6

**Figure 2:**
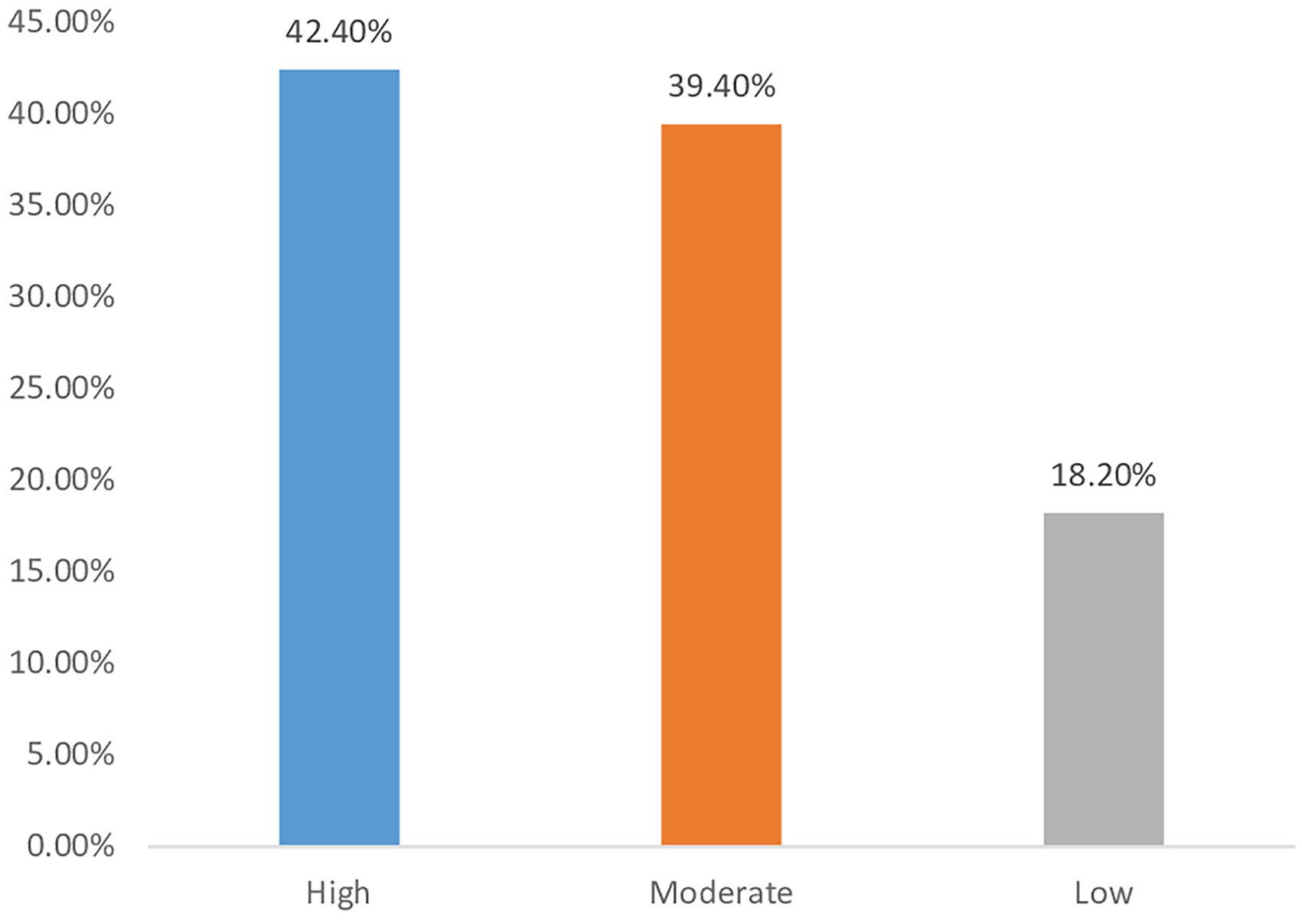
Overall level of knowledge of participants about COVID-19.

Figure 2 shows the overall level of knowledge respondents had on COVID-19 which was measured on a scale of high, medium and low level. From this figure, 42.40% of respondents had a high level of knowledge, 39.40% scored moderately, and 18.20% had low level of knowledge.

### 3.3 Participants attitudes toward COVID-19 vaccine

Table 4 illustrates participants’ attitudes toward the vaccine. On investigating the importance of discussing risks and benefits of the vaccine with healthcare givers if pregnant, planning to conceive or breastfeeding; a majority of 34.3% agreed, 27.3% strongly agreed, 25.3% were neutral, 11.1% disagreed and 2.0% strongly disagreed. Regarding advising their family and friends to get vaccinated, 9.1% strongly agreed, 21.2% agreed, 40.4% were neutral, 17.2% disagreed, and 12.1% strongly disagreed. When asked if they could accept the vaccine in future if required for traveling purposes, 53.5% said yes while 46.5% said no.

**Table 4:** Participants attitudes toward COVID-19 vaccine.

Variable	Modality	Frequency	Percentage
Discuss risks/benefits of the vaccine with healthcare worker if pregnant, planning to conceive	Strongly agree	54	27.3
	Agree	68	34.3
	Neutral	50	25.3
	Disagree	22	11.1
	Strongly disagree	4	2.0
Receive next COVID-19 dose while breastfeeding	Strongly agree	20	10.1
	Agree	32	16.2
	Neutral	90	45.5
	Disagree	34	17.2
	Strongly disagree	22	11.1
Advice family and friends to be vaccinated.	Strongly agree	18	9.1
	Agree	42	21.2
	Neutral	80	40.4
	Disagree	34	17.2
	Strongly disagree	24	12.1
Receive vaccine if required for travel purposes?	Yes	106	53.5
	No	92	46.5

### 3.4 Participants beliefs about COVID-19 vaccine

Table 5 shows the participants beliefs about COVID-19 vaccine. When asked if the vaccine should not be given to older adults, 7.1% strongly agreed, 19.2% agreed, 38.4% were neutral, 25.3% disagreed, and 10.1% strongly disagreed. Upon finding out if preventive measures could stop after receiving first dose of the vaccine, 9.1% strongly agreed, 24.2% agreed, 25.3% were neutral, 23.2% disagreed, and 18.2%strongly disagreed. On finding out from participants what measures can be taken to address COVID-19 vaccine refusal in the community, 29.3% said vaccination should be made mandatory, 61.6% said it will not be necessary to force people, while 9.1% said unvaccinated persons should be isolated from the community. Additionally, on finding out whether COVID-19 vaccination was promoted for financial reasons, 20.2% strongly agreed, 17.2% agreed, 36.4% were neutral, 20.2% disagreed and 6.1% strongly disagreed.

**Table 5:** Participants beliefs about COVID-19 vaccine.

Variable	Modality	Frequency	Percentage
COVID-19 vaccine should not be given to older adults	Strongly agree	14	7.1
	Agree	38	19.2
	Neutral	76	38.4
	Disagree	50	25.3
	Strongly disagree	20	10.1
Stop following other preventive measures after first dose?	Strongly agree	18	9.1
	Agree	48	24.2
	Neutral	50	25.3
	Disagree	46	23.2
	Strongly disagree	36	18.2
Measures taken to address vaccine refusal and hesitancy?	Mandatory	58	29.3
	Not necessary to force people	122	61.6
	Isolate unvaccinated people	18	9.1
COVID-19 vaccination promoted for financial reasons?	Strongly agree	40	20.2
	Agree	34	17.2
	Neutral	72	36.4
	Disagree	40	20.2
	Strongly disagree	12	6.1
COVID-19 vaccines are harmful to women planning to conceive?	Strongly agree	34	17.2
	Agree	62	31.3
	Neutral	86	43.4
	Disagree	12	6.1
	Strongly disagree	4	2.0

### 3.5 Prevalence of COVID-19 vaccine acceptance

**Figure 3:**
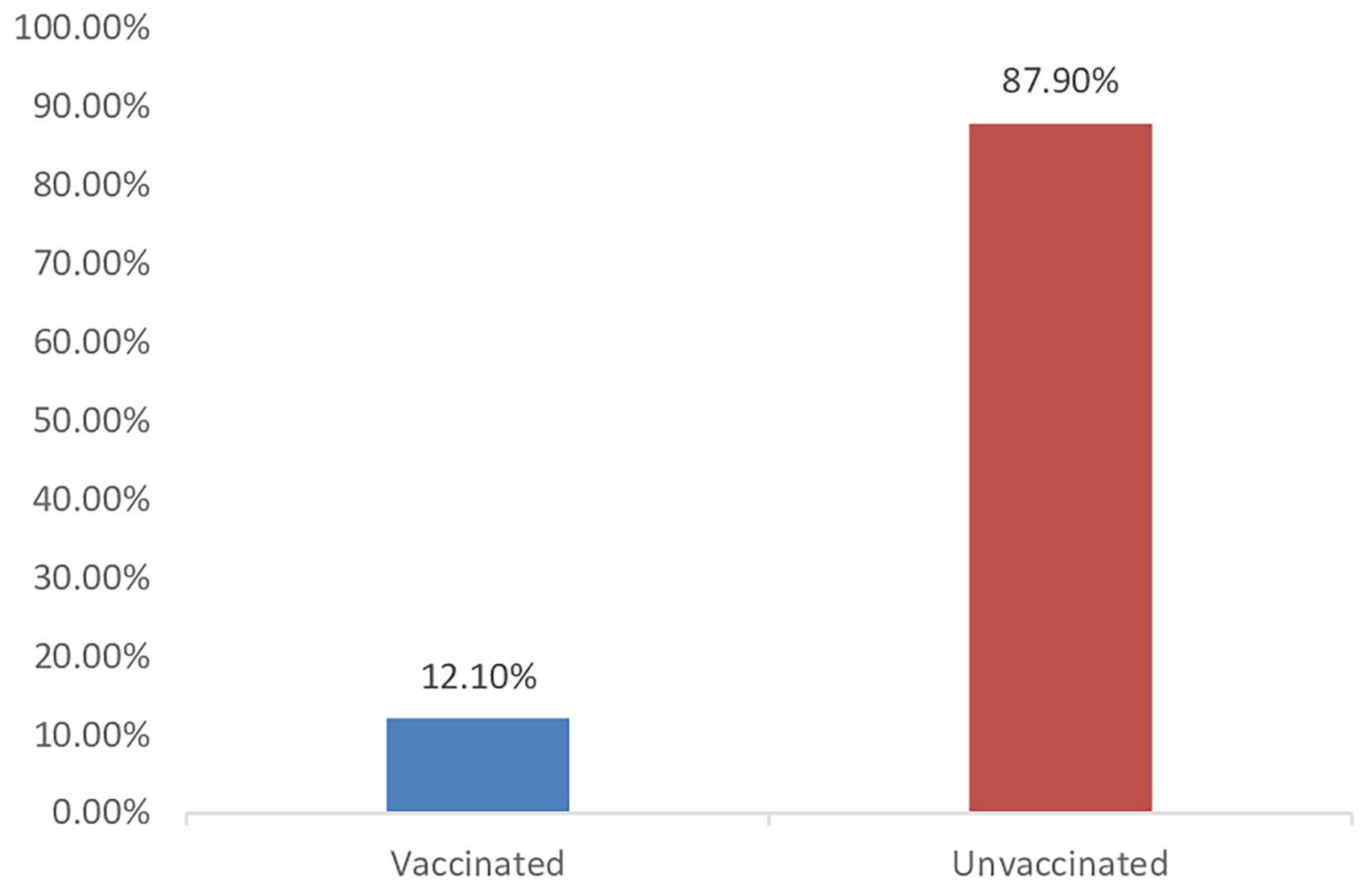
Overall percentage of vaccinated and unvaccinated persons.

Figure 3 shows the overall percentage of vaccinated and unvaccinated persons in Bamenda I during the peak of the pandemic. It follows from the analysis of this figure that, 12.10% of the study population were vaccinated, while 87.90% were unvaccinated.

### 3.6 COVID-19 vaccine refusal with respect to sociodemographic factors

Table 6 shows the association between sociodemographic factors and COVID-19 vaccine refusal. The analysis revealed that age, gender, and educational background were significantly associated with vaccine refusal (*p* < 0.05), indicating that these factors may influence individuals’ decisions regarding COVID-19 vaccination. In contrast, profession did not show a statistically significant association with vaccine refusal (*p* > 0.05), suggesting that occupational status may not be a determining factor in this context.

**Table 6:** COVID-19 vaccine refusal with respect to socio-demographic factors.

Variable	Modality	Receive the COVID-19 vaccine		Chi-square	*p*-value
		Vaccinated (%)	Unvaccinated (%)		
Age	15–20	6(3.03)	82(41.41)	**10.59**	**0.014**
	21–30	8(4.04)	58(29.29)		
	31–40	6(3.03)	28(14.14)		
	40 and above	4(2.02)	6(3.03)		
Gender	Male	16(8.08)	78(39.39)	**4.03**	**0.045**
	Female	8(4.04)	96(48.48)		
Education	Primary	2(1.01)	2(1.01)	**16.04**	**0.001**
	Secondary	10(5.05)	42(21.21)		
	University	6(3.03)	110(55.55)		
	Not educated	6(3.03)	20(10.10)		
Profession	Entrepreneur	6(3.03)	34(17.17)	3.661	0.300
	Health personnel	4(2.02)	14(7.07)		
	Student	8(4.04)	90(45.45)		
	Others	6(3.03)	36(18.18)		

### 3.7 COVID-19 vaccine refusal with respect to knowledge of participants

Table 7 illustrates the association between participants’ knowledge and specific reasons for COVID-19 vaccine refusal. The analysis indicates that factors such as concerns about vaccine safety, doubts about its effectiveness, perceptions of alternative preventive measures, and public health considerations were all significantly associated with vaccine refusal (*p* < 0.05). These findings suggest that both individual knowledge levels and specific perceived risks play a critical role in shaping attitudes toward COVID-19 vaccination.

**Table 7:** Association between knowledge and reasons for COVID-19 vaccine refusal.

Variable	Modality	Received the COVID-19 vaccine		Chi-square	*p*-value
		Vaccinated (%)	Unvaccinated (%)		
Virus responsible for COVID-19?	Ebola	0(0)	4(2.02)	1.453	0.484
	Corona	24(12.12)	164(82.82)		
	Hepatitis A	0(0)	6(3.03)		
	Herpes simplex	0(0)	0(0)		
Awareness of COVID-19 vaccination center nearby?	Yes	8(4.04)	32(16.16)	2.925	0.232
	No	6(3.03)	52(26.26)		
	I do not know	10(5.05)	90(45.45)		
Main reason for vaccine refusal?	Vaccine may be dangerous	14(7.07)	54(27.27)	**15.538**	**0.001**
	Parents refused	2(1.01)	20(10.10)		
	Religion forbids me	4(2.02)	8(4.04)		
	I do not have a reason	4(2.02)	92(46.46)		
Concerned about the safety of the vaccine?	Yes	12(6.06)	144(72.72)	**15.265**	**0.002**
	No	12(6.06)	30(15.15)		
Believed in the effectiveness of the vaccine?	Yes	20(10.10)	68(34.34)	**16.728**	**0.000**
	No	4(2.02)	106(53.53)		
COVID-19 is a serious public health threat?	Yes	10(5.05)	138(69.69)	**15.834**	**0.000**
	No	14(7.07)	36(18.18)		
Some preventive measures against COVID-19?	Covid 19 vaccine	8(4.04)	26(13.13)	**15.786**	**0.003**
	Hand washing	4(2.02)	22(11.11)		
	Nose masks	8(4.04)	26(13.13)		
	Herbal remedies	0(0)	28(14.14)		
	All of above	4(2.02)	72(36.36)		
Was COVID-19 vaccine development was rushed?	Yes	10(5.05)	90(45.45)	2.044	0.563
	No	14(7.07)	84(42.42)		
Encountered COVID-19 vaccine misinformation?	Yes	14(7.07)	92(46.46)	0.253	0.615
	No	10(5.05)	82(41.41)		

### 3.8 COVID-19 vaccine refusal with respect to attitudes

Table 8 presents the association between participants’ attitudes and COVID-19 vaccine refusal. The results indicate that variables such as the importance of discussing the risks and benefits of receiving the COVID-19 vaccine with a healthcare provider and concerns about receiving the next COVID-19 dose while breastfeeding were significantly associated with vaccine refusal (*p* < 0.05). These findings highlight the role of healthcare communication and reproductive health considerations in shaping vaccine-related decisions.

**Table 8:** Association between participant’s attitudes toward COVID-19 vaccine.

Variable	Modality	Received the COVID-19 vaccine		Chi-square	*p*-value
		Vaccinated (%)	Unvaccinated (%)		
COVID-19 vaccine is harmful to women planning to conceive?	Strongly agree	2(1.01)	32(16.16)	**31.152**	**0.000**
	Agree	8(4.04)	54(27.27)		
	Neutral	8(4.04)	78(39.39)		
	Disagree	2(1.01)	10(5.05)		
	Strongly disagree	4(2.02)	0(0)		
Discuss risks/benefits of COVID-19 vaccine with healthcare worker?	Strongly agree	6(3.03)	48(24.24)	1.507	0.825
	Agree	8(4.04)	60(30.30)		
	Neutral	8(4.04)	42(21.21)		
	Disagree	2(1.01)	20(10.10)		
	Strongly disagree	0(0)	4(2.02)		
Receive next dose of vaccine while breastfeeding?	Strongly agree	8(4.04)	12(6.06)	**24.579**	**0.000**
	Agree	0(0)	32(19.16)		
	Neutral	12(6.06)	78(39.39)		
	Disagree	0(0)	34(17.17)		
	Strongly disagree	4(2.02)	18(9.09)		
Advise family and friends to be vaccinated?	Strongly agree	0(0)	18(9.09)	8.155	0.086
	Agree	6(3.03)	36(18.18)		
	Neutral	14(7.07)	66(33.33)		
	Disagree	4(2.02)	30(15.15)		
	Strongly disagree	0(0)	24(12.12)		
Receive vaccine if required for traveling purposes?	Yes	16 (8.08)	88(44.4)	2.329	0.312
	No	8(4.04)	86(43.43)		

### 3.9 COVID-19 vaccine refusal with respect to beliefs

Table 9 shows the association between participant’s beliefs and COVID-19 vaccine variables such as COVID-19 vaccine should not be given to older adults, after receiving the first COVID-19 vaccine dose should other preventive measures. Beliefs such as; COVID-19 vaccine is harmful to women planning to conceive, stop following preventive measures after receiving the vaccines, and COVID-19 vaccine should not be given to older adults were significantly associated with COVID-19 vaccine refusal.

**Table 9:** Association between participant’s beliefs and COVID-19 vaccine refusal.

Variable	Modality	Received the COVID-19 vaccine		Chi-square	*p*-value
		Vaccinated (%)	Unvaccinated (%)		
COVID-19 vaccine should not be given to older adults	Strongly agree	4(2.02)	10(5.05)	**17.1**	**0.002**
	Agree	6(3.03)	32(16.13)		
	Neutral	8(4.04)	68(34.34)		
	Disagree	0(0)	50(25.25)		
	Strongly disagree	6(3.03)	14(7.07)		
Stop following preventive measures after receiving first dose	Strongly agree	10(5.05)	8(4.04)	**41.4**	**0.000**
	Agree	8(4.04)	40(20.20)		
	Neutral	4(2.02)	46(23.23)		
	Disagree	0(0)	46(23.23)		
	Strongly disagree	2(1.01)	34(17.17)		
Measures taken to address vaccine refusal and hesitancy	Mandatory	6(3.03)	52(26.26)	0.3	0.860
	Not necessary to force people	16(8.08)	106(53.53)		
	Isolate unvaccinated people	2(1.01)	16(8.08)		
COVID-19 promoted for financial reasons	Strongly agree	6(3.03)	34(17.17)	0.7	0.940
	Agree	4(2.02)	30(15.15)		
	Neutral	8(4.04)	64(32.32)		
	Disagree	4(2.02)	36(18.18)		
	Strongly disagree	2(1.01)	10(5.05)		

## 4 Discussion

Merely 12.10% of people in Bamenda I had received the COVID-19 vaccination. The majority of participants consisted of young people, with 33.3% of the respondents being in their 20s and 30s. Although people aged 20–30 generally have stronger immune systems and are less likely to die from COVID-19, they can still become seriously ill, be hospitalized, or suffer long-term effects (long COVID). The vaccine helps reduce: risk of severe symptoms, risk of hospitalization and risk of long COVID (29). This result is in line with earlier research emphasizing the important impact of age on COVID-19-related knowledge, attitudes, and actions (30). The younger population may have different perceptions of the virus’s risk and susceptibility, which may have an impact on their preventive measures (31). Our results also showed a significant association between age and unwillingness to get the COVID-19 vaccination which is consistent with a study conducted on COVID-19 vaccine hesitancy in peri-urban areas in Kanpur, Uttar Pradesh, India (32). The acceptance of COVID-19 vaccines has demonstrated substantial regional disparities, with reported rates ranging from 36% in Africa to 83% in Oceania (33). Globally, vaccine hesitancy rates have varied considerably, estimated between 23.6 and 97% (34, 35). Within Africa, hesitancy prevalence has been reported at 42.2% in Uganda (36), 53.9% in Ethiopia (37), and 48% across 16 countries on the continent (38). Nigeria reports a hesitancy rate of 49.8% (39), while Arab countries exhibit a rate of 37.6% (40). Outside the African region, the prevalence of vaccine hesitancy includes 19.1% in Canada (41), 20% in both Mexico and India (38), 21.4% in other Indian studies (42), 25.5% in Bangladesh (43), and 33% in Italy (44). These findings emphasize that vaccine hesitancy remains a globally heterogeneous phenomenon, influenced by geographical context (45). Certain demographic groups are disproportionately affected by vaccine hesitancy. Higher rates have been observed among women, individuals with lower educational levels, low-income populations, Black adults, those with comorbid conditions, and residents of rural or semi-urban areas (43). Younger individuals often believe they are less likely to get seriously ill or die from COVID-19 (46). This leads to a lower perceived personal benefit from getting vaccinated, reducing motivation. This contrasts with a study conducted in Venezuela that reported no significant association (47), potentially due to differences in the age groups studied. Additionally, 52.5% of participants were female, indicating a slight gender imbalance favoring women.

Women have generally shown higher COVID-19 vaccination rates than men in many countries, and this trend can be explained by a combination of biological, social, behavioral, and cultural factors. Women are more likely than men to seek healthcare proactively, attend regular checkups, and follow public health advice. This includes being more willing to accept vaccinations as a form of preventive care. Research suggests that gender disparities can influence COVID-19 knowledge and behaviors, with women generally showing greater awareness and commitment to preventive measures (48). The relationship between gender and vaccine refusal in our study was significant, differing from Terry et al.’s (49) findings, which indicated no significant association. This discrepancy may be attributed to the heightened health related concerns and susceptibility to vaccine misinformation often observed among women. Misinformation about the COVID-19 vaccine causing infertility may also contribute to these differences. Regarding education, most respondents (26.3%) had completed secondary school, while 58.6% held university degrees and 13.1% were uneducated. A significant association was found between education level and vaccine refusal.

People with higher education levels often have better health literacy, meaning they are more likely to: understand medical information, assess the risks and benefits of vaccines, recognize misinformation (50). This makes them more likely to trust vaccines and understand their importance. Consistent with other studies on vaccine hesitancy (51), people with higher achievement of education were not as likely to exhibit vaccine reluctance compared to those with lower education levels. Educated individuals are more likely to access information from various sources, enabling them to make informed decisions compared to those with limited literacy skills. Education also helps to dispel misconceptions and cultural practices surrounding health. However, this contrasts with findings from a mixed-methods study in Senegal, which found no meaningful correlation between education and Coronavirus vaccine refusal (52). This discrepancy may stem from the fact that about 97% of Senegalese are Muslim, and there may be a cultural context that de-emphasizes education.

In our study, the occupational distribution revealed that 49.5% of respondents were students. The fact that 49.5% were students suggests that students represent a significant portion of the vaccine-hesitant population in the studied group. This finding is significant, as students particularly those in healthcare fields can play a vital role in raising awareness about COVID-19 and promoting preventive measures within their communities (53).

Our results indicated that most participants had high (42.40%) to moderate (39.40%) levels of knowledge about COVID-19, consistent with a study conducted in Menoua Division, Cameroon, which also found high knowledge levels among participants (28). This similarity may be attributed to greater exposure to COVID-19-related information. However, our findings contrast with those from a study in Venezuela, which reported low to moderate levels of knowledge about the virus, potentially due to inadequate communication and access to vaccination campaigns there (47).

Additionally, our study revealed that many respondents were unaware of nearby COVID-19 centers, with 20.2% indicating knowledge of such facilities. This is inconsistent with the Menoua Division study, where 58.6% of respondents were aware of local vaccination centers (28). This discrepancy might be related to the retrospective nature of our study, as the pandemic’s decline may have led people to forget specifics about vaccination availability. Lack of knowledge regarding healthcare resources can hinder timely diagnosis, treatment, and preventive measures, underscoring the need for enhanced communication and outreach efforts to inform the public about available COVID-19 services.

According to our study, individuals who were immunized against COVID-19 had a greater understanding of the illness and its prevention methods. Specifically, they exhibited higher knowledge levels about COVID-19 and were more aware of the vaccination clinics nearby. In contrast, the unvaccinated group showed a lower comprehension of vaccination and otherpreventative actions, such using masks and washing your hands. Given that evidence shows that vaccination intentions and behaviors are strongly influenced by faith in the safety and efficacy of vaccinations, this knowledge gap could represent a substantial obstacle to vaccine uptake (28, 53).

Our findings indicated that a significant majority (87.90%) of participants were unvaccinated against COVID-19, with 12.10% having received the vaccine. This aligns with the study by Tetsatsi et al. (28), which reported a similar vaccination rate of 10%. In contrast, vaccination acceptance rates were notably higher in Burkina Faso and Nigeria, at 66.53 and 74.47%, respectively, where common reasons for refusal included perceived lack of necessity, safety concerns, and parental opposition. This hesitancy is concerning, as it may facilitate the virus’s further propagation and exacerbate risks for vulnerable populations. Notably, about 26% of women expressed willingness to receive the vaccine while breastfeeding, likely due to widespread misinformation regarding vaccine safety, such as fears about infertility, as noted by Emily Terry et al. (54). The reasons for vaccine hesitancy-safety concerns, perceived ineffectiveness, and exposure to misinformation-are consistent with previous studies on vaccine attitudes and behaviors (55). Addressing these issues through targeted educational campaigns and fostering trust in scientific and public health authorities will be essential for improving COVID-19 vaccination rates and enhancing future pandemic preparedness (51).

In our study, we found a meaningful correlation age group and refusal of the COVID-19 vaccination, with many participants believing that vaccines should not be administered to older adults. This contrasts with the findings of Tetsatsi et al. (28), who noted that the older population is more vulnerable to the disease and therefore more likely to accept vaccination. This discrepancy may stem from our respondents’ belief that older individuals are naturally immune to infections. Additionally, other beliefs observed in our study included the perception that COVID-19 vaccination serves as a means for financial exploitation and the notion that individuals could discontinue all preventive measures after receiving the first dose of the vaccine. However, these beliefs are less common in other studies, making direct comparisons challenging.

## 5 Conclusion

This study which was aimed at unveiling the factors associated with COVID-19 vaccine refusal in Bamenda I sub-division of the Northwest Region had indicated that the COVID-19 vaccine refusal was substantially correlated with socio-demographic factors like age, gender, and educational attainment. Our findings revealed that while most participants were familiar with COVID-19, their knowledge of other diseases like Ebola and Hepatitis A was limited. Regarding attitudes toward the COVID-19 vaccination, many women engaged in discussions with health workers about the risks and benefits. Participants held several negative beliefs and myths about the vaccines.

### 5.1 Limitations

This study has several limitations that should be acknowledged. First, as a retrospective study relying on self-reported data, it is subject to recall bias, as participants were asked to remember their attitudes and behaviors during the height of the COVID-19 pandemic. Additionally, the use of questionnaires and self-administered surveys may have introduced social desirability bias, potentially affecting the accuracy of responses. The sample size, while adequate for preliminary analysis, may limit the generalizability of the findings to the broader population. Furthermore, the retrospective cross-sectional design does not allow for causal inferences between variables.

### 5.2 Future research

Future research should consider qualitative approaches, such as in-depth interviews and focus group discussions, to explore underlying beliefs, cultural influences, and emotional factors driving vaccine refusal. Longitudinal studies could also help track changes in attitudes over time and evaluate the effectiveness of targeted interventions aimed at improving vaccine acceptance in similar settings.

## Data Availability

The original contributions presented in the study are included in the article/supplementary material, further inquiries can be directed to the corresponding authors.
